# Molecular pain, a new era of pain research and medicine

**DOI:** 10.1186/1744-8069-1-1

**Published:** 2005-01-14

**Authors:** Jianguo Gu, Min Zhuo, Michael Caterina, Amy B MacDermott, Annika Malmberg, Volker Neugebauer, Megumu Yoshimura

**Affiliations:** 1 Department of Oral & Maxillofacial Surgery, McKnight Brain Institute and College of Dentistry, University of Florida, Gainesville, Florida 32610, USA; 2 Department of Physiology, Faculty of Medicine, University of Toronto, Toronto, Ontario M5S 1A8, Canada; 3 Department of Biological Chemistry, Johns Hopkins School of Medicine, Baltimore, Maryland 21205, USA; 4 Department of Physiology and Cellular Biophysics, Columbia University, 630 West 168th Street, New York, NY 10032, USA; 5 Elan Pharmaceuticals, 800 Gateway Boulevard, San Francisco, CA 94080, USA; 6 Department of Neuroscience and Cell Biology, The University of Texas Medical Branch, 301 University Blvd. RT 1069, Galveston, TX 77555-1069, USA; 7 Department of Integrative Physiology, Graduate School of Medical Sciences, Kyushu University, Maidashi 3-1-1, Higashiku, Fukuoka 812-8582, Japan

## Abstract

Molecular pain is a relatively new and rapidly expanding research field that represents an advanced step from conventional pain research. Molecular pain research addresses physiological and pathological pain at the cellular, subcellular and molecular levels. These studies integrate pain research with molecular biology, genomics, proteomics, modern electrophysiology and neurobiology. The field of molecular pain research has been rapidly expanding in the recent years, and has great promise for the identification of highly specific and effective targets for the treatment of intractable pain. Although several existing journals publish articles on classical pain research, none are specifically dedicated to molecular pain research. Therefore, a new journal focused on molecular pain research is needed. *Molecular Pain*, an Open Access, peer-reviewed, online journal, will provide a forum for molecular pain scientists to communicate their research findings in a targeted manner to others in this important and growing field.

## 

The word pain is thought to derive from the Latin word poena, meaning punishment. An emotional reaction to a punishment might have been what Aristotle experienced, as he defined pain as an emotional event. René Descartes, the seventeenth-century philosopher and scientist, pictured a pain pathway consisting of a thread with two ends: one end is in a peripheral part of the body, for example a toe, and the other end is a bell in the brain. According to this picture, fire touching a toe pulls the thread, and rings the bell to sound a warning in the brain. Over the past decades, and in the current Decade of Pain Control and Research (2001–2010), pain research has undergone major changes, from a system level to cellular, subcellular and molecular levels. A new era of molecular pain research is now emerging, and the journal *Molecular Pain *is dedicated to this modern phase of pain research.

Recent advances in pain research are in large part due to the rapid progress in neuroscience, molecular biology, and other fields in the life sciences. Breakthroughs in biomedical technologies have allowed us to address many important issues about pain, enriching our knowledge about the mechanisms by which sensory signals including pain are initiated, encoded, conducted, transmitted, modulated, and perceived. For example, sensory molecular biology has led to the molecular cloning and identification of a number of receptors involved in thermal, mechanical, and nociceptive signalling at the periphery, some of which have been targeted for pain management. Modern electrophysiology has been used to demonstrate the critical roles of synaptic plasticity in pain processing in the spinal cord and the brain. Long-term potentiation and long-term depression at synapses of central sensory regions have delineated the 'memory of pain' by neuronal circuitry along pain transmitting pathways. Functional imaging of supraspinal areas has revealed central areas related to pain processing (for example, areas coding behavioural learning and memory) and, more significantly, it has now become possible to see the alteration of these signalling pathways under chronic pain conditions. Finally, genomics and proteomics have been applied to pain research to help identify the changes in the array of molecules present in cells under chronic pain conditions. Research within all these fields will provide a better understanding of the physiological and pathological mechanisms of pain.

Pain research at the cellular, subcellular, and molecular levels has provided insights that help guide the treatment and management of intractable pain conditions including neuropathic pain, cancer pain and other chronic pain conditions. At the same time, these efforts continue to provide scientific insights into an inherently fascinating biological process. New molecules related to pain continue to be cloned and identified. It should be stressed that a 'non-pain molecule' under physiological conditions can become a 'pain molecule' under pathological conditions. This might be an underlying mechanism for spontaneous pain or pain sensation elicited by innocuous stimuli. Thus, the aims of molecular pain research should include 'non-pain molecules'. One big task facing us is that many of the pain-related genes or proteins that have been identified are also important for other neuronal functions in the spinal cord and the brain. Pain triggers various responses in the spinal cord and the brain, including reflexes, conscious perception, cognitive learning and memory processes, emotional reaction such as depression, and drug addiction. Thus, molecules that are associated with pain are not only those located on the peripheral nerve endings for the sensing and encoding of stimuli, but also molecules that are present along sensory paths from the spinal cord to the brain for integrating and modulating sensory information. Molecular targets at different levels along sensory pathways are key to future identification of new drugs and therapies that effectively manage intractable pain conditions with low side effects. Molecular pain research will offer new opportunities for drug development in the pharmaceutical industry and improved treatment options in the clinical setting.

In our journey to explore pain mechanisms and to identify effective targets for pain management, it is important for scientists around the world to have a rapid and freely accessible forum for exchanging ideas, debating hot topics, developing collaborations, promoting science, and improving pain medicine. As an online, Open Access journal, *Molecular Pain *will help to fulfil these goals. The journal's Open Access policy changes the way in which articles are published. Firstly, all articles become freely and universally accessible online, and so an author's work can be read by anyone at no cost. Secondly, the authors hold copyright for their work and grant anyone the right to reproduce and disseminate the article, provided that it is correctly cited and no errors are introduced. Thirdly, a copy of the full text of each Open Access article is permanently archived in an online repository separate from the journal. *Molecular Pain*'*s *articles are archived in PubMed Central (), the US National Library of Medicine's full-text repository of life science literature, and also in repositories at the University of Potsdam () in Germany, at INIST () in France and in e-Depot (), the National Library of the Netherlands' digital archive of all electronic publications.

The launch of *Molecular Pain *would not have been possible without the strong support of many neuroscientists who have carried out pain-related research for many years. *Molecular Pain *has a strong editorial board with wide expertise in pain-related research, from peripheral sensory receptors to cortical sensory processing centers. *Molecular Pain*'s editorial board members have committed to support the journal by helping identify important and interesting research manuscripts, serving as reviewers, and directly contributing their work to the journal. We aim to publish papers in a timely fashion. Each manuscript will be peer-reviewed by two experts, and the review process is anticipated to be completed within three weeks. Once accepted, papers will be published online immediately, and they will be listed in PubMed as soon as possible after publication. We hope that *Molecular Pain *will become a high impact journal, a journal that provides new directions for pain research and medicine, and a home for creative scientists. We welcome researchers and clinicians as readers of and contributors to, *Molecular Pain*.

## Competing interests

The author(s) declare that they have no competing interests.

